# The Impact of Botulinum Toxin Injection on the Outcomes of Breast Surgeries: A Systematic Review and Meta-Analysis

**DOI:** 10.1007/s00266-023-03466-0

**Published:** 2023-07-18

**Authors:** Abdelrahman Awadeen, Mohamed Fareed, Ali Mohamed Elameen

**Affiliations:** 1 Department of Plastic and Reconstructive Surgery, Faculty of Medicine (Boys), Al-Azhar University, Al Mokhaym Al Daem, Gameat Al Azhar, Nasr City, Cairo, Egypt; 2 Department of Plastic and Reconstructive Surgery, El-Sahel Teaching Hospital, Cairo, Egypt

**Keywords:** Botulinum toxin, Breast, Pectoralis major, Animation deformity

## Abstract

**Background:**

Breast surgeries aim to restore the natural appearance of the breasts with acceptable functional and cosmetic outcomes. However, these surgical procedures may be associated with considerable adverse events. The present systematic review and meta-analysis was designed to reveal the functional and aesthetic outcomes of botulinum toxins (BTX) injection in patients subjected to breast surgeries.

**Methods:**

A literature review was performed up to 21 September 2022. All clinical studies included patients older than 18 years old and treated with BTX injection for breast surgeries were included.

**Results:**

The present study included 12 articles, encompassing 496 patients. The average dosage of BTX injection ranged from 20 to 100 units. Injecting BTX significantly reduced the mean post-operative opioid analgesics usage (SMD −1.577; 95% −2.087, −1.067; *P* < 0.001) and the risk of severe animation deformity (RR 12.37; 95% 1.76, 86.66; *P* = 0.01). There was a statistically significant higher mean expansion volume per visit in the BTX injection group (SMD 1.166; 95% 0.313, 2.018; *P* = 0.007). There was no statistically significant impact of BTX injection on the risk of surgical site infection (RR 0.59; 95% 0.15, 2.34; *P* = 0.45) and seroma (RR 0.51; 95% 0.03, 10.15; *P* = 0.66).

**Conclusions:**

The present study revealed the potential benefits of BTX injection in breast surgeries. This included reduced post-operative analgesics, as well as the risk of severe animation deformity. This was accomplished with increased expansion volume per visit and a similar risk of BTX injection-related complications.

**Level of Evidence III:**

This journal requires that authors assign a level of evidence to each article. For a full description of these Evidence-Based Medicine ratings, please refer to the Table of Contents or the online Instructions to Authors www.springer.com/00266.

**Supplementary Information:**

The online version contains supplementary material available at 10.1007/s00266-023-03466-0.

## Introduction

The female breasts represent a prominent secondary sexual characteristic. In addition to the physiological role in breastfeeding, breasts are associated culturally with fertility and womanhood [1, 2]. Importantly, more than 2.5 million women yearly diagnosed with breast cancer in the US. Breast-conservative surgeries are the commonest surgical interventions for patients with early breast cancer. Meanwhile, approximately 110,000 women subjected to breast reconstruction yearly [3–5]. Aesthetic breast surgeries are among the most performed cosmetic surgical procedures worldwide. More than 500,000 patients are subjected to aesthetic breast surgeries in the US annually. Augmentation mammoplasty was the commonest procedure, contributing to approximately 60% of all aesthetic breast surgeries [6, 7].

Breast surgeries aim to restore the natural appearance of the breasts with acceptable functional and cosmetic outcomes. However, these surgical procedures may be associated with considerable adverse events [8, 9]. Manipulation of the pectoralis muscles, placement of the breast implants, and subsequent filling of the expanders may be associated with negative repercussions. Chronic post-operative pain is encountered among 50% of patients subjected to aesthetic breast surgeries. This pain is mainly attributable to the prolonged spasm and the abnormal involuntary contraction of the pectoralis major muscle [10, 11]. This contraction in the mammary region may lead to significant breast deformities. This included high breast riding, implant dislocation, formation of tethering bands, and capsular contracture. Excessive proliferation of the fibroblasts at the wound site results in pathological scars. These scars are associated with pain and itching and may result in functional and cosmetic morbidities. These consequences decrease patients’ satisfaction, prolong hospital stays, and delay the time needed for definitive reconstruction [12–14]. Subsequently, extensive spasms of the pectoralis major muscle may lead to premature removal of breast expanders [15]. The desire for women to recreate aesthetic breasts with fewer post-operative complications highlighted the need to prevent abnormal muscle contraction after breast surgeries. Denervation of the pectoralis major muscle by blocking median and lateral pectoral nerves may provide a solution. Paradoxically, the inconsistent anatomy of nerves in the mammary region makes the denervation procedures challenging and risky [16].

Botulinum toxin (BTX) is a neurotoxin that blocks the release of acetylcholine at the nerve terminals. This resulted in chemical denervation of the muscles, decreasing the muscular spasms. Injection of BTX has been widely used for treating patients with painful muscle spasms such as fibromyalgia, temporomandibular joint dysfunction, and cervical dystonia [17–19]. Reducing abnormal pectoralis major muscle activity in the setting of breast surgeries should maintain the desired cosmetic outcomes while preventing functional impairment. Whereas many published studies revealed the usability and safety of BTX injection for reducing abnormal muscle activity, the functional and cosmetic results in breast surgeries deserved further assessment [20–22]. Herein, this systematic review and meta-analysis study was designed to summarize the functional and aesthetic outcomes of BTX injection in patients subjected to breast surgeries

## Methods

This systematic review was executed parallel with PRISMA guidelines [23], and the recommendations of the Cochrane collaboration [24]**. **The methodology of the present systematic review was registered at PROSPERO database (CRD42022360780). (Supplementary Table.1)

### Data Source

A systematic searching of the literature was performed up to 21 September 2022. The following databases were searched: Google Scholar, PubMed, Scopus, Web of Science, NYAM, VHL, SIGLE, Clinical trials, mRCT, EMBASE, ICTRP, and Cochrane Collaboration. The searching implemented no restrictions to the patients’ age, ethnicity, sex, date of publication, language of publication, or place of the studies.

Each database was searched based on controlled vocabulary words. A manual searching of the references of the eligible articles was performed to recognize all non-indexed additional studies. The following keywords were used; ‘Oncoplastic’, ‘Mammoplasty’, ‘Reconstruction’, ‘Reduction’, ‘Augmentation’, ‘Breast’, ‘Pectoral’, ‘Pectoralis’, ‘Mammaplasty’, ‘Botulinum’, ‘Abobotulinum’, ‘Botox’, ‘Dysport’, ‘Neurotoxin’.

### Eligibility Criteria

All clinical studies included patients older than 18 years old and treated with BTX injection for breast surgeries were eligible for systematic review. Non-human studies, overlapped data, review articles, case reports, guidelines, comments, erratum, letters, book chapters, editorials, and meeting abstracts were excluded. The screening of title, abstract, and full-texts of the relevant articles was executed blindly by two authors to detect the eligible studies for data extraction. Controversial findings were dissolved by discussion with the third author. The flow chart revealed the details of the screening and eligibility processes.

### Data Extraction

The study characteristics data were revealed from the eligible studies. This included the title of the included study, study identification, study design, registration number, study region, eligibility criteria, and study period. Baseline patients’ characteristics were revealed. This included the age of the patients, sample size, race, and ethnicity. The variables related to breast procedures were revealed, which included the type of breast of surgery, the timing of reconstruction, implant size, pocket location of the implant, intra-operative filling amount, implant type, and implant coverage. Furthermore, the stage of breast cancer, previous mastectomy, and history of previous therapies were revealed. The BTX injection-related data were extracted, including the dosage, timing and route of administration, and times of injections. The effectiveness and safety of BTX injection were assessed based on post-operative pain at different intervals, post-operative analgesics use, number of expansion sessions, time to final breast procedure, and scar-related variables. The complications related to BTX injection were evaluated. This included the risk of surgical site infection, seroma, and skin necrosis. Extracting the data from graphs was performed using WebPlotDigitizer software [25]. Two authors extracted the data independently using Microsoft excel sheet.

### Risk of Bias and Quality Assessment

The Cochrane Collaboration's tool for assessing the risk of bias was used for evaluating the bias risk in the included randomized clinical trials (RCTs) [26]. The National Institute of Health (NIH) quality assessment tool was used to assess the quality of the eligible retrospective and prospective clinical studies [27].

### Statistical Analysis

Standardized mean difference (SMD) or weighted mean difference (WMD) was used for pooling the continuous data. Data reported in the form of mean and range or median and range were converted to mean and standard deviation (SD) [28]. The risk ratio (RR) and their 95% confidence interval (95%CI) was used for reporting the dichotomous outcomes. The fixed-effect model was used when the homogeneity between the eligible studies was revealed. Conversely, the random-effects model was used. Statistical heterogeneity was assessed using Higgins *I*^*2*^ statistic, at the value of > 50%, and the Cochrane Q (*Chi*^2^ test), at the value of *P*  <  0.10 [29]. Data analysis was performed using Review Manager version 5.4 and Comprehensive Meta-Analysis v3 software [30, 31]. The significance was established when the result of probability value (*P*) < 0.05.

## Results

**Fig. 1:**
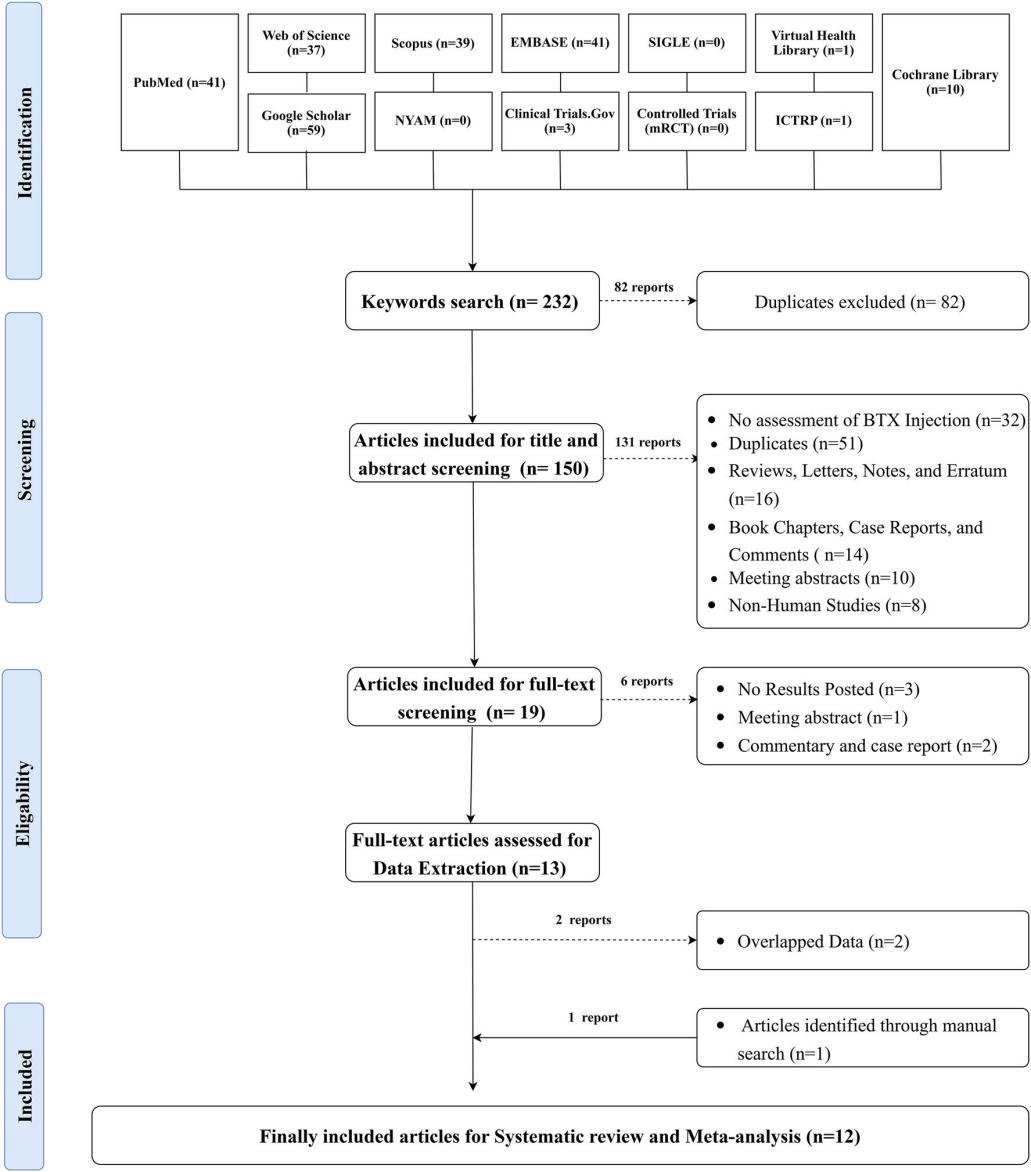
PRISMA Flow chart showing the process of the literature search, title, abstract, and full text screening, systematic review, and meta-analysis

Searching the literature resulted in 232 articles. Of them, 150 articles were eligible for the title and abstract screening, excluding 82 duplicates. Consequently, 131 articles were eliminated, revealing 19 eligible for full-text screening. Six studies were ousted, yielding 13 studies eligible for data extraction. Two articles with overlapped data were excluded, resulting in 11 studies eligible for data extraction. One article was included in the manual search, resulting in 12 articles eligible for systematic review and meta-analysis. The search approach for the included databases is revealed in Supplementary Table 2. The screening processes are shown in the PRISMA flowchart. **(**Fig. 1)

### Demographic Characteristics of the Eligible Studies

This systematic review and meta-analysis study included 12 articles [32–43]. Five articles were RCTs, while three were retrospective designs. Of the included 496 patients, 333 were treated with BTX injection. The average age of the eligible patients ranged from 32 to 56.6 years. Breast reconstruction was carried out among 350 patients. Whereas 83 patients underwent a mastectomy, 43 patients received breast-conservative surgeries (Table 1).

**Table 1:** Demographic characteristics of the included studies

Study ID		Study region	Study design	Study period	Demographic characteristics					Type of breast surgery	
					Sample size		Total sample Size	Age (years)		Mastectomy	
					Botulinum	Control		Botulinum	Control	Botulinum	Control
					Number	Number	Number	Mean ±SD	Mean ±SD	Number	Number
1	Abedini et al., 2020[32]	Iran	RCT	October 2018 to December 2019	19	19	19	37.84 ± 7.1	0	0	0
2	Disphanurat et al., 2021[35]	Thailand	RCT	November 2019 to January 2020	22	22	22	NR	NR	0	0
3	Ermilova et al., 2020[36]	Russia	Prospective case-control study	NR	38	34	72	32*	0	0	0
4	Figus et al., 2009 [37]	Italy	Prospective study	January 2002 to April 2006	13	13	13	NR	NR	0	0
5	Gabriel et al., 2015[38]	USA	Prospective study	January to December 2008	15	15	30	44.5 ( 27–64)*	0	0	0
6	Greof et al., 2018[34]	Belgium	RCT	February 2015 and July 2016	25	25	50	53. ±10.0	56.6±10.0	12	17
7	Iriarte et al., 2012[33]	Spain	Quasi-experimental study	June 2009 and February 2011	33	33	33	NR	NR	33	33
8	Layeeque et al., 2004[39]	USA	Retrospective study	July 2001 and November 2003	22	26	48	55±11	52±10	0	0
9	Lemaine et al., 2020[40]	USA	RCT	May 2012 and May 2017	68	63	131	49.9 ±11.1	48.4 ±11.5	0	0
10	Lo et al., 2015 [41]	USA	RCT	October 2009 to February 2012	23	23	23	46.5 (28 –68)*	0	0	0
11	Ma et al., 2021[42]	USA	Retrospective study	2012 to 2019	7	7	7	51.5 ( 34–73)*	0	0	0
12	Puentes-Gutiérrez et al., 2021[43]	Spain	Retrospective study	January 2018 to January 2020,	48	48	48	NR	NR	21	0

Study ID		Type of breast surgery									
		Mastectomy with immediate reconstruction		Breast conservative surgeries		Mammoplasty		Breast reconstruction		Prosthesis	
		Botulinum	Control	Botulinum	Control	Botulinum	Control	Botulinum	Control	Botulinum	Control
		Number	Number	Number	Number	Number	Number	Number	Number	Number	Number
1	Abedini et al., 2020[32]	0	0	0	7	7	0	0	6	6	
2	Disphanurat et al., 2021[35]	0	0	0	0	22	22	0	0	0	0
3	Ermilova et al., 2020[36]	0	0	0	0	0	38	34	0	0	
4	Figus et al., 2009 [37]	0	0	0	0	0	0	13	13	NR	NR
5	Gabriel et al., 2015[38]	0	0	0	0	0	15	15	0	0	
6	Greof et al., 2018[34]	3	2	10	6	0	0	0	0	0	0
7	Iriarte et al., 2012[33]	0	0	0	0	0	0	0	0	0	0
8	Layeeque et al., 2004[39]	0	0	0	0	0	0	22	26	0	0
9	Lemaine et al., 2020[40]	0	0	0	0	0	0	68	63	0	0
10	Lo et al., 2015 [41]	0	0	0	0	0	23	23	0	0	
11	Ma et al., 2021[42]	0	0	0	0	0	48	48	0	0	
12	Puentes-Gutiérrez et al., 2021[43]	0	27	0	0	0	0	0	0		

*RCT* randomized controlled trial, *NR* non-reported, *SD* standard deviation

^*^Data reported in the form of median and range, ** data reported in the form of mean only

The average dosage of BTX injection ranged from 20 to 100 units. Five articles implemented BTX injection during breast surgeries, while one article implemented it before the surgery. Pectoralis major muscle was the main site of BTX injection. The average follow-up duration ranged from two weeks to 36 months (Table 2).

**Table 2:** Intervention, implant related data, and quality assessment of the included studies

Study ID		Botulinum toxin injection related data					Control group	Implant type
		Dosage	Timing of injection	Site of injection	Root of administration	Times of Injection		
1	Abedini et al., 2020[32]	50 units of incobotulinumtoxin -A prepared with 1 ml of 0.9% normal saline (50 IU/ml)	5–10 days after surgery	to the either side of scars	Intradermally	1 Time	Pre-Treatment	NR
2	Disphanurat et al., 2021[35]	20 units (0.5 mL) of BTX-A	Immediately after skin closure	along the 4-cm long wound	Intradermally	1 Time	Contralateral non-treated side	NR
3	Ermilova et al., 2020[36]	100 units on each side in different administered 1:25 ml	two weeks prior surgery	Pectoralis major muscle	Intramuscular injection	1 Time	0.9% NaCl solution	Silicone implants
4	Figus et al., 2009 [37]	100 U of BTX-A diluted with 5 cc of normal saline solution	After operation	Reconstructed breast	Percutaneous injection	2–3 Times	Pre-Treatment	Silicone cohesive gel
5	Gabriel et al., 2015[38]	BTX-A (2 cc of 20 units/mL)	During operation	Pectoralis major muscle surrounding the prosthesis	Intramuscular injection	1 Time	2 cc of 0.9% NaCl	NR
6	Greof et al., 2018[34]	BTX-A (100 units)	After operation	Pectoralis major muscle	Intramuscular injection	1 Time	physical therapy and 1 saline infiltration	NR
7	Iriarte et al., 2012[33]	100 units of Botox® or 250 of Dysport® diluted in 4ml of normal saline solution	During the filling phase of the expander or after prosthesis	Pectoralis major muscle	Intramuscular injection	1–2 times	Pre-treatment	NR
8	Layeeque et al., 2004[39]	100 units of BTX-A diluted in 40 to 60 mL of normal saline	During operation	Pectoralis major, serratus anterior, rectus abdominis	Intramuscular injection	1 Time	Untreated	NR
9	Lemaine et al., 2020[40]	100 units of BTX-A and reconstituted in 0.9% sodium chloride	During operation	Pectoralis major muscle	Intramuscular injection	1 Time	5 mL of 0.9% sodium chloride	NR
10	Lo et al., 2015 [41]	100 U of BTX-A diluted in 10 mL of normal saline	During operation	Pectoralis major muscle	Intramuscular injection	1 Time	Normal saline	Saline-based
11	Ma et al., 2021[42]	100 unit BTX-A vial reconstituted with 5 mL of sterile sodium chloride (0.9%).	After operation	Pectoralis major muscle and latissimus dorsi muscle	Intramuscular injection	1 Time	NR	NR
12	Puentes-Gutiérrez et al., 2021[43]	100 units (U) of onabotulinumtoxin-A or 250 U of abobotulinum-A	During operation	Pectoralis major muscle	Intramuscular injection	1 Time	Pre-Treatment	NR

Study ID		Implant size (mL)		Plane of implant	Follow-up period	Quality assessment	
		Botulinum	Control				
		Mean ±SD	Mean ±SD		Months	%	Decision
1	Abedini et al., 2020[32]	NR	NR	NR	2 weeks, 3 months, and 6 months	–	–
2	Disphanurat et al., 2021[35]	NR	NR	NR	3 month, 6-month, and 9-month	–	–
3	Ermilova et al., 2020[36]	295 up to 415 ml		Subpectoral	Two weeks	66.66%	Good
4	Figus et al., 2009 [37]	Expander 145–465 prostheses 145–370	NA	Subpectoral	(1, 3, 6 and 12 months)	75%	Good
5	Gabriel et al., 2015[38]	250–400	250–400	NR	every 1-3 months for 23.4 (12-36 months)	75%	Good
6	Greof et al., 2018[34]	NR	NR	NR	6 Months	–	–
7	Iriarte et al., 2012[33]	NR	NR	NR	1, 3, and 6 months	66.66%	Good
8	Layeeque et al., 2004[39]	535 ± 144	526 ± 165	Subpectoral	2 years	66.66%	Good
9	Lemaine et al., 2020[40]	NR	NR	Subpectoral	NR	–	–
10	Lo et al., 2015 [41]	Expander Size 250 to 550		Subpectoral	12 weeks		–
11	Ma et al., 2021[42]	NR	NR	NR	8.5 (8–24 months)	66.66%	Good
12	Puentes-Gutiérrez et al., 2021[43]	NR	NR	NR	6 weeks	66.66%	Good

*BTX* botulinum toxin, *NaCL* sodium chloride, *NR* non-reported, *SD* standard deviation

### Risk of Bias and Quality Assessment

**Fig. 2:**
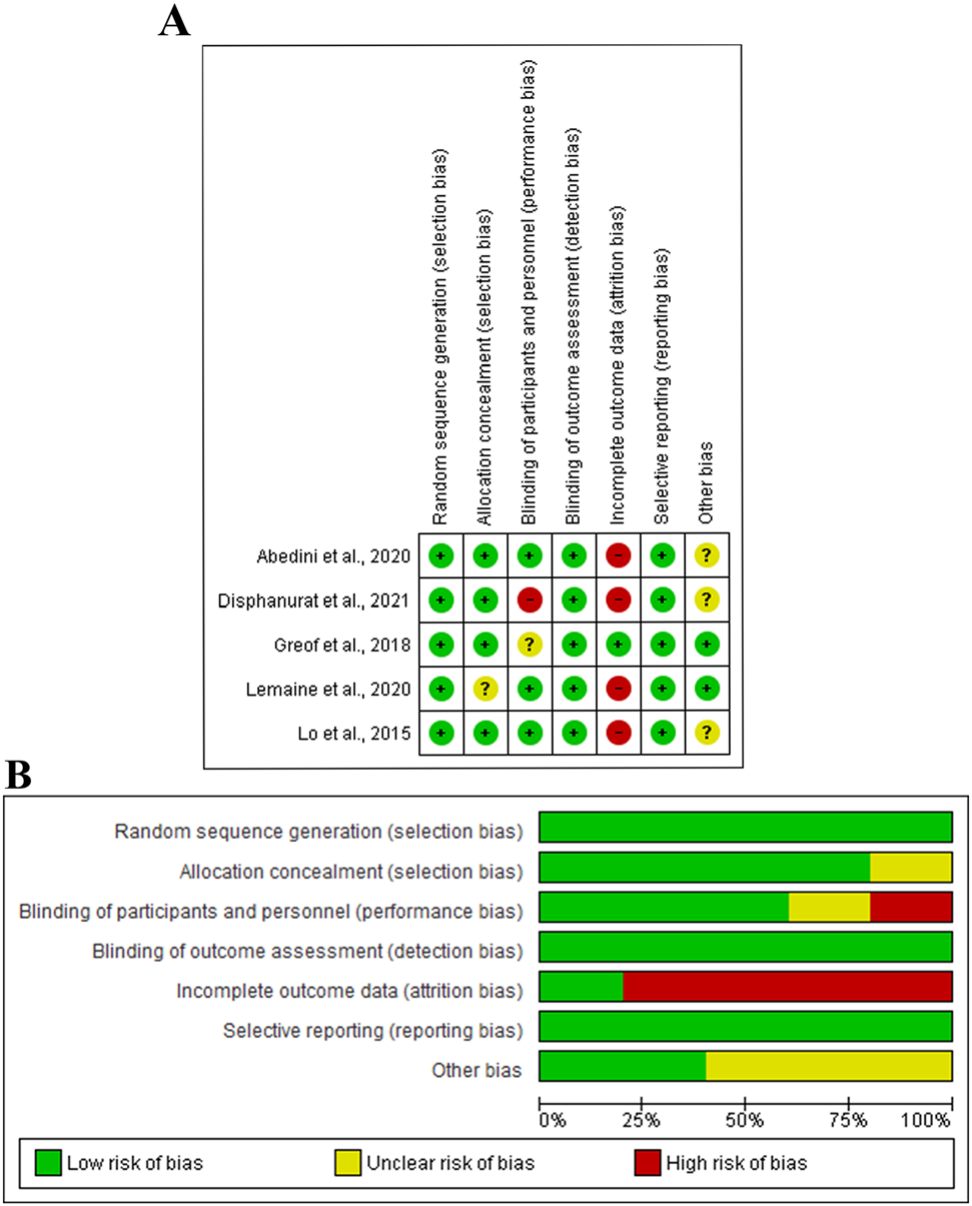
**A** Risk of bias graph, **B** Risk of bias summary: review authors' judgements about each risk of bias item presented as percentages across all included studies

All the included RCTs [32, 34, 35, 40, 41] showed a low risk of detection, selection, and reporting biases. Lamaine et al., [40] showed an unclear risk of selection bias, while Disphanurat et al., [35] showed high risk of performance bias. The included observational retrospective and prospective studies were of good quality (Fig. 2 and Table 2).

### Study Outcomes

#### Post-Operative Pain

##### Immediate Post-Operative Pain

**Fig. 3:**
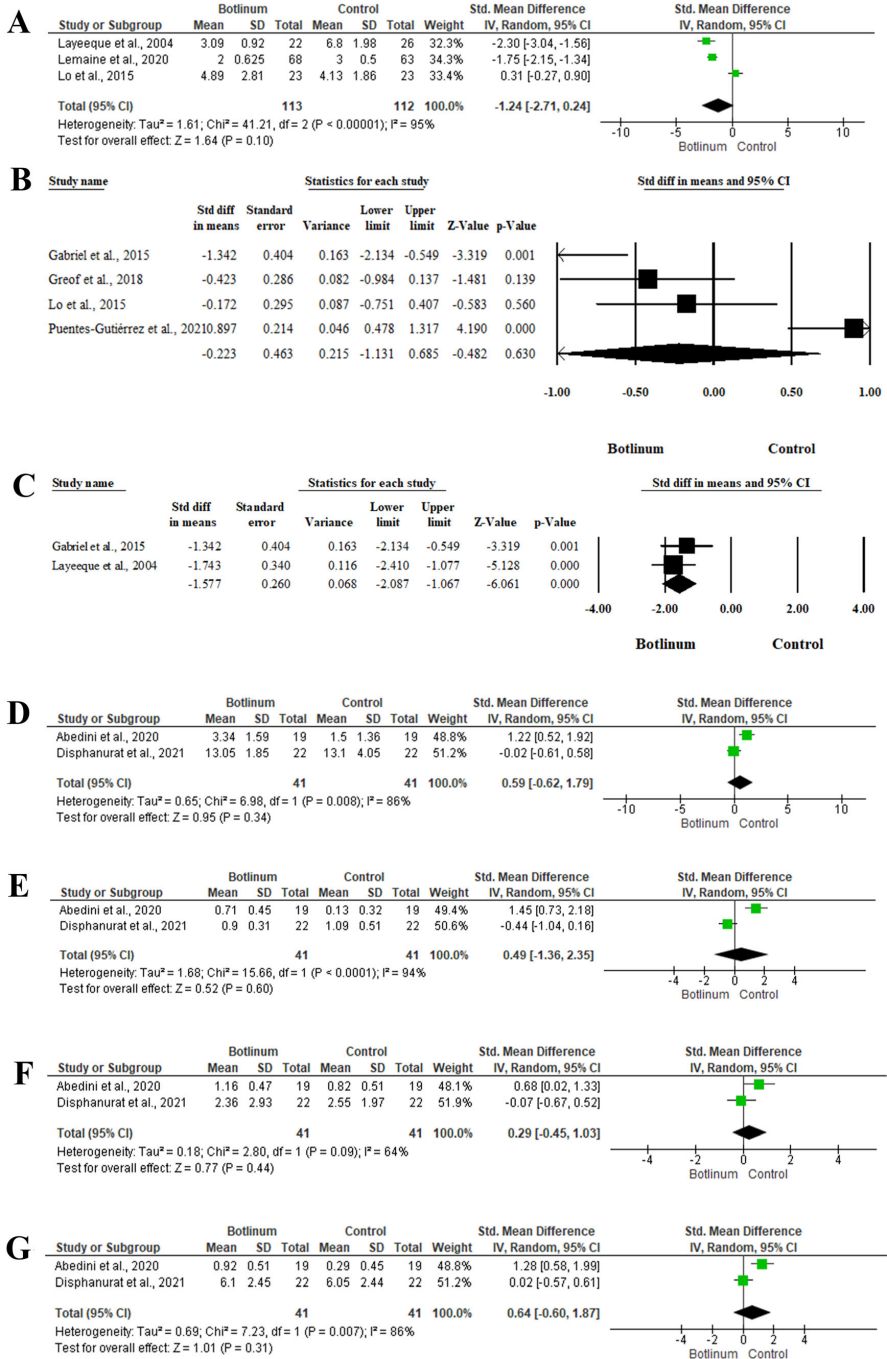
Forest plot of summary analysis of the (**A**) Standardized Mean Difference (SMD) and 95% CI of mean immediate post-operative pain between BTX injection and control groups (** B**) Standardized Mean Difference (SMD) and 95% CI of mean post-operative pain at last follow-up period between BTX injection and control groups (** C**) Standardized Mean Difference (SMD) and 95% CI of the mean opioid analgesic use between BTX injection and control groups (** D**) Standardized Mean Difference (SMD) and 95% CI of the overall scar evaluation at three months between BTX injection and control groups (**E**) Standardized Mean Difference (SMD) and 95% CI of the mean scar width at three months between BTX injection and control groups. (**F**) Standardized Mean Difference (SMD) and 95% CI of the mean scar height at three months between BTX injection and control groups. (**G**) Standardized Mean Difference (SMD) and 95% CI of the mean scar colour at three months between BTX injection and control groups. Size of the green or and black squares is proportional to the statistical weight of each trial. The grey diamond represents the pooled point estimate. The positioning of both diamonds and squares (along with 95% CIs) beyond the vertical line (unit value) suggests a significant outcome (IV = inverse variance)

Three studies [39–41] included 202 patients and evaluated the mean immediate post-operative pain scores between BTX injection and control groups. Pooling the effect sizes revealed no significant impact of BTX injection on the immediate post-operative pain (SMD −1.24; 95% −2.71, 0.24; *P* = 0.10) with heterogeneity between the included studies (*I*^2^ = 95%, *P*<0.001) (Fig. 3A).

##### Post-Operative Pain at the Last Follow-up

The mean post-operative pain at the last follow-up between BTX injection and control groups was reported in four articles [34, 38, 41, 43], including 135 patients. There was no significant impact of BTX injection on the mean post-operative pain at the last follow-up interval (SMD −0.223; 95% −1.131, 0.685; *P* = 0.630) using the random-effects model (*I*^2^ = 90.041%, *P* < 0.001). (Fig. 3B)

##### Opioid Analgesics Use

Two studies [38, 39] included 53 patients reported the mean opioid analgesic use between BTX injection and control groups. In the random-effects model (*I*^2^ = 0%, *P* = 0.447), patients who treated with BTX injection showed a statistically significant lower mean post-operative opioid analgesics usage, in comparison to the control group (SMD −1.577; 95% −2.087, −1.067; *P* < 0.001). The control groups received 2 cc of 0.9% NaCl and untreated in the Gabriel et al and Layeeque et al., respectively [38, 39] (Fig. 3C).

#### Post-Operative Scar Evaluation

##### At Three-Months

Two studies [32, 35], including 41 patients, evaluated post-operative scar after breast surgeries. There was no significant impact of BTX injection on the overall scar evaluation (SMD 0.59; 95% −0.62, 1.79; *P* = 0.34). Meanwhile, there was no significant difference between BTX injection and control groups in the terms of scar width (SMD 0.49; 95% −1.36, 2.35; *P* = 0.60), scar height (SMD 0.29; 95% −0.45, 1.03; *P* = 0.44), and scar colour (SMD 0.64; 95% −0.60, 1.87; *P* = 0.31) (Fig 3D–G).

##### At Six-Months

**Fig. 4:**
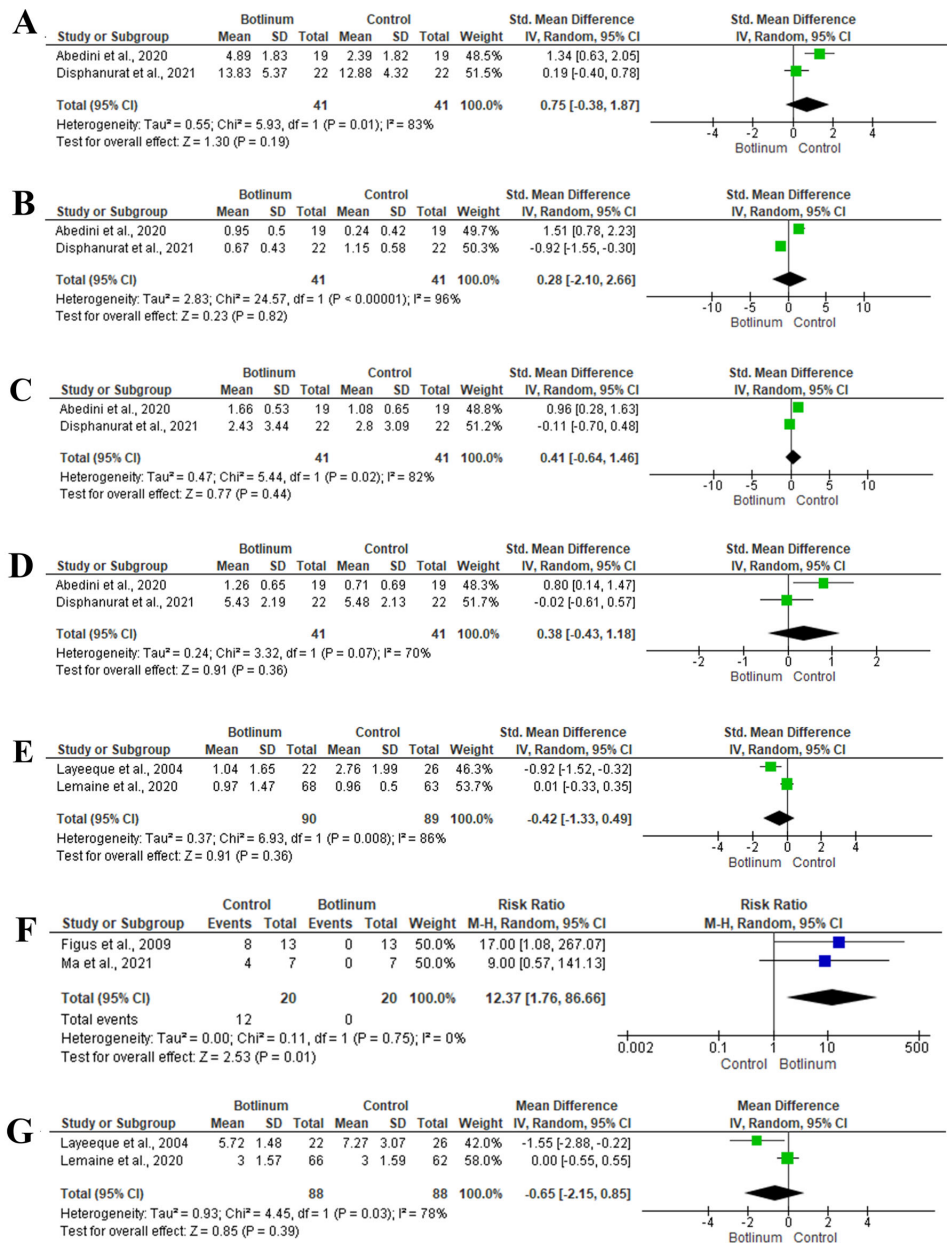
Forest plot of summary analysis of the** A** Standardized Mean Difference (SMD) and 95% CI of the overall scar evaluation at six months between BTX injection and control groups **B** Standardized Mean Difference (SMD) and 95% CI of the mean scar width at six months between BTX injection and control groups **C** Standardized Mean Difference (SMD) and 95% CI of the mean scar height at six months between BTX injection and control groups **D** Standardized Mean Difference (SMD) and 95% CI of the mean scar colour at six months between BTX injection and control groups **E** Standardized Mean Difference (SMD) and 95% CI of the mean pain levels during the expansion process between BTX injection and control groups **F** The Risk ratio (RR) and 95% CI of the risk of severe animation deformity between BTX injection and control groups **G** Mean Difference (MD) and 95% CI of the mean number of expansion sessions between BTX injection and control groups. Size of the green, blue, or and black squares is proportional to the statistical weight of each trial. The grey diamond represents the pooled point estimate. The positioning of both diamonds and squares (along with 95% CIs) beyond the vertical line (unit value) suggests a significant outcome (IV = inverse variance)

The impact of BTX injection on the outcomes of post-operative scar at six months was revealed in two articles [32, 35], including 41 patients. There was no significant difference between BTX injection and the control group regarding the overall scar evaluation (SMD 0.75; 95% −0.38, 1.87; *P* = 0.19) and scar width (SMD 0.28; 95% −2.10, 2.66; *P* = 0.82). There was no significant impact of BTX injection on the scar height (SMD 0.29; 95% −0.45, 1.03; *P* = 0.44) and scar colour (SMD 0.64; 95% −0.60, 1.87; *P* = 0.31) (Fig 4A–D).

#### Expansion Process and Animation Deformity

##### Pain During the Expansion Process

Two studies [39, 40], including 179 patients, assessed the mean pain level during the expansion process. Pooling the data revealed no significant impact of BTX injection on the mean pain level during the expansion process (SMD −0.42; 95% −1.33, 0.49; *P* = 0.36). (Fig. 4E)

##### Animation Deformity

Two studies [37, 42], including 20 patients, showed the impact of BTX injection on the risk of severe animation deformity. The risk of severe animation deformity was 12.37 times more in the control group, in contrast to patients in the BTX injection group (RR 12.37; 95% 1.76, 86.66; *P* = 0.01). (Fig. 4F)

##### Number of Expansion Sessions

The mean number of expansion sessions between BTX injection and control groups was reported in two articles, including 176 patients [39, 40]. There was no statistical difference between both groups (SMD −0.65; 95% −2.15, 0.85; *P* = 0.39) with significant heterogeneity between the included articles (*I*^2^ = 78%, *P* = 0.03). (Fig. 4G)

##### Expansion Volume per Visit

**Fig. 5:**
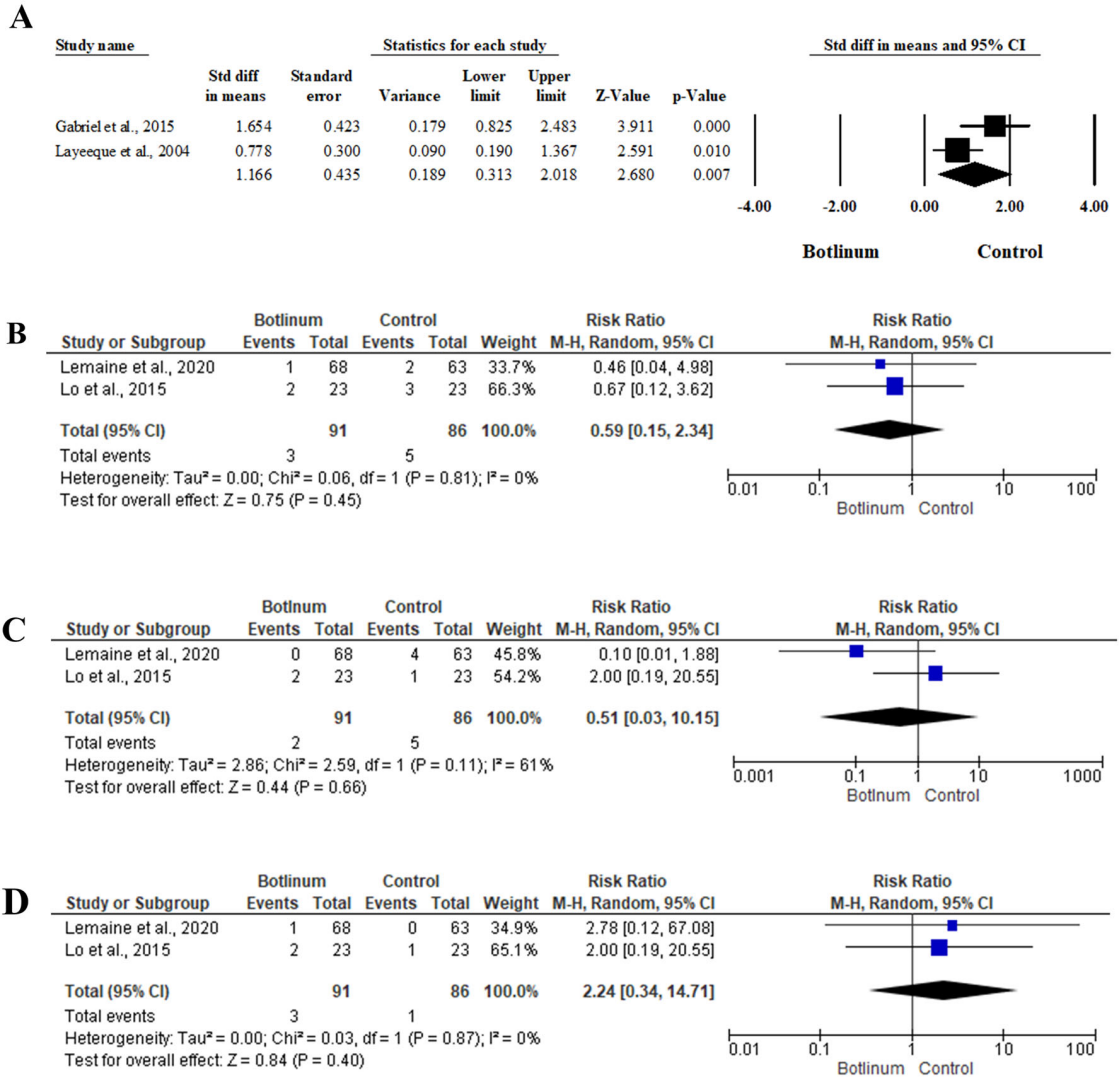
Forest plot of summary analysis of the **A** Standardized Mean Difference (SMD) and 95% CI of the mean expansion volume per visit between BTX injection and control groups **B** The Risk ratio (RR) and 95% CI of the risk of surgical site infection between BTX injection and control groups **C** The Risk ratio (RR) and 95% CI of the risk of seroma between BTX injection and control groups **D** The Risk ratio (RR) and 95% CI of the risk of skin necrosis between BTX injection and control groups. Size of the black or blue squares is proportional to the statistical weight of each trial. The grey diamond represents the pooled point estimate. The positioning of both diamonds and squares (along with 95% CIs) beyond the vertical line (unit value) suggests a significant outcome (IV = inverse variance)

Two studies [38, 39] included 63 patients and assessed the mean expansion volume per visit between BTX injection and control groups. There was a statistically significant higher mean expansion volume per visit in the BTX injection group (SMD 1.166; 95% 0.313, 2.018; *P* = 0.007) in the random-effects model (*I*^2^ = 64.9%, *P* = 0.091) (Fig. 5A).

#### Post-Operative Complications

Two studies [40, 41] included 154 patients reported the safety of BTX injection for patients with breast surgeries. There was no significant impact of BTX injection on the risk of surgical site infection (RR 0.59; 95% 0.15, 2.34; *P* = 0.45), seroma (RR 0.51; 95% 0.03, 10.15; *P* = 0.66), and skin necrosis (RR 2.24; 95% 0.34, 14.71; *P* = 0.40). (Fig. 5B–D)

## Discussion

There is a paucity of evidence regarding the effectiveness and safety of BTX injection in the settings of breast surgeries. Therefore, the present systematic review and meta-analysis was carried out to reveal the functional and aesthetic outcomes of BTX injection in breast surgeries. The current study showed that injection of BTX significantly reduced post-operative opioid analgesics usage, minimized the risk of severe animation deformity, and increased the expansion volume per visit. There was no significant impact of BTX injection on the post-operative pain level and post-operative scar evaluation. The safety of BTX injection has been established with a similar risk of surgical site infection, seroma, and skin necrosis as the non-injected group.

Post-operative pain after breast surgeries is an unavoidable sequence. In the present systematic review, injecting BTX neither significantly reduced immediate post-operative pain nor pain during the expansion process. This finding was parallel with Li et al., [20] systematic review. They reported the ineffective role of BTX injection in controlling the pain after placing the implants deeper, within the pectoralis major muscle, in patients subjected to mammaplasty. On the contrary, Winocour et al., [22] proposed a significant role of BTX injection in alleviating post-operative pain associated with implants and expanders placement in the subpectoral plane. Importantly, BTX injection have analgesic and paralytic properties by reversibly inhibiting the release of neurotransmitters at the presynaptic neuromuscular junction. Injection of BTX inhibits the release of neuropeptides, reducing the inflammatory reaction and minimizing pain sensation. However, these effects occur within seven days of injection, reach the peak within 14 days, and last for several weeks [42, 44, 45]. This might explain ineffective role of BTX in reducing the immediate post-operative pain levels. Furthermore, there was a significant increase in the amount of post-operative opioid analgesics used among the control group to provide pain control comparable to the BTX injection group.

Post-operative scarring after breast surgeries is a major therapeutic challenge. Whereas scarless breast surgeries are difficult to be achieved, injecting BTX has been proposed to minimize the post-operative scar. In the present systematic review, there was no significant impact of intradermal injection of BTX on the scar width, height, and colour in the short and long terms. Contradictory to this finding, Wang et al., [46] reported the beneficial role of BTX injection in treating facial scares, with significant improvement in scar width. Yue et al., [47] revealed that BTX injection improves the cosmetic appearance of facial scars by reducing the risk of hyperplasia. Of note, BTX injection regulates scar formation by controlling the expression of α-smooth muscle actin and inhibiting fibroblast-to-myofibroblast differentiation [48]. The tension on the wound edges is a critical mechanism for scar formation. The resultant forces activate local fibroblasts to induce collagen and transforming growth factors expression. Injecting BTX causes temporary muscle paralysis, reduces the tension at the wound edges, and minimizes scar development [49]. However, the ineffective role of BTX in reducing scar development in breast surgeries might be attributable to the more significant tension load on the skin of the breasts and trunk relative to the face. Furthermore, intradermal injection of BTX did not induce paralysis of the underlying pectoralis major muscle. The thick subcutaneous fat at the inframammary fold act as a barrier for BTX diffusion to the underlying muscles. Contraction of the pectoralis major muscle produces tension at the wound edge, resulting in mechanical tension perpendicular to the incision and increasing the risk of hypertrophic scars [50].

The expansion volume per visit was significantly increased after BTX injection. This was accomplished with a significantly decreased risk of severe animation deformity. Relaxing the pectoralis muscles allowed more fluid to be inserted into the expanders in each visit, shortening the time needed for definitive reconstruction. Paradoxically, the expansion volume may be affected by the implant size, type, pocket location, and patients’ characteristics. Further studies are needed to assess the impact of BTX injection on the time necessary for definitive reconstruction in the context of these factors. The reduction of the pectoralis muscle tone accomplished more satisfactory and natural breast contour. However, high dosages of BTX injection may extend to the adjacent muscles, resulting in adverse paralysis. Furthermore, the clinical effects of BTX injection are temporary and may require repeated injections to accomplish a long-term effect [21, 51].

The present systematic review gathered the available evidence related to the safety and efficacy of BTX injection for breast surgeries. On the contrary, there were some limitations to be considered in clinical practice. There was significant heterogeneity between the eligible studies regarding the protocol of BTX injection. This included a difference in the dosage, type, site, timing, times, and route of BTX administration. Further studies are required to detect the safest and the most effective protocol of BTX injection in the settings of breast surgeries. Statistical heterogeneity was revealed with the assessed outcomes. Such heterogeneity may be attributed to the variation in study designs, demographic characteristics, injection protocols, and follow-up periods. The random-effects model was implemented to mitigate this heterogeneity. Furthermore, the long-term effects of BTX injection on the outcomes of breast surgeries need to be investigated.

## Conclusions

The present study revealed the off-label potential benefits of BTX injection in breast surgeries. This included reduced post-operative analgesics, as well as the risk of severe animation deformity. This was accomplished with increased expansion volume per visit and a similar risk of BTX injection-related complications. There was no significant impact of BTX injection on the post-operative scar evaluation. Further RCTs with long-term follow-up periods are required to alleviate the limitations of the current study.

### Supplementary Information

Below is the link to the electronic supplementary material.

Supplementary file1 (DOCX 31 KB)

## References

[1] Milothridis P, Pavlidis L, Haidich A-B, Panagopoulou E (2016). A systematic review of the factors predicting the interest in cosmetic plastic surgery. Indian J Plas Surg.

[2] Montemurro P, Cheema M, Hedén P (2018). Patients’ and surgeons’ perceptions of social media’s role in the decision making for primary aesthetic breast augmentation. Aesthetic Surge J.

[3] Jonczyk MM, Jean J, Graham R, Chatterjee A (2019). Surgical trends in breast cancer: a rise in novel operative treatment options over a 12 year analysis. Breast Cancer Res Treat.

[4] American Society of Plastic Surgeons (2018) Plastic Surgery Statistics Report

[5] Fraser VJ, Nickel KB, Fox IK, Margenthaler JA, Olsen MA (2016). The epidemiology and outcomes of breast cancer surgery. Trans Am Clin Climatol Assoc.

[6] Statistics CSNDB (2018). Cosmetic surgery national data bank statistics. Aesthetic Surg J.

[7] Gupta V, Yeslev M, Winocour J, Bamba R, Rodriguez-Feo C, Grotting JC, Higdon KK (2017). Aesthetic breast surgery and concomitant procedures: incidence and risk factors for major complications in 73,608 cases. Aesthetic Surg J.

[8] Zhang L, Zheng J, Mu J, Gao Y, Li G (2022). Risk factors for postoperative complications following aesthetic breast surgery: a retrospective cohort study of 4973 patients in China. Aesthetic Plast Surg.

[9] Kim JY (2021) Managing Common and Uncommon Complications of Aesthetic Breast Surgery. Springer, Cham

[10] Gärtner R, Jensen M-B, Nielsen J, Ewertz M, Kroman N, Kehlet H (2009). Prevalence of and factors associated with persistent pain following breast cancer surgery. JAMA.

[11] Oliver JD, Knackstedt R, Gatherwright J (2020) Optimizing pain control after implant-based breast reconstruction: a systematic review. Plast Reconstruct Surg Global Open 8(7 Suppl) 10.1080/2000656X.2020.1800480 32734796

[12] Fischer JP, Wes AM, Nelson JA, Basta M, Rohrbach JI, Wu LC, Serletti JM, Kovach SJ (2014). Propensity-matched, longitudinal outcomes analysis of complications and cost: comparing abdominal free flaps and implant-based breast reconstruction. J Am College Surg.

[13] Malahias M, Jordan D, Hughes L, Hindocha S, Juma A (2016). A literature review and summary of capsular contracture: an ongoing challenge to breast surgeons and their patients. Int J Surg Open.

[14] Slatman J, Halsema A, Meershoek A (2016). Responding to scars after breast surgery. Qual Health Res.

[15] Disa JJ, Ad-El DD, Cohen SM, Cordeiro PG, Hidalgo DA (1999). The premature removal of tissue expanders in breast reconstruction. Plast Reconstruct Surg.

[16] Blanco R (2011). The ‘pecs block’: a novel technique for providing analgesia after breast surgery. Anaesthesia.

[17] Naumann M, Albanese A, Heinen F, Molenaers G, Relja M (2006). Safety and efficacy of botulinum toxin type A following long-term use. Europ J Neurol.

[18] Thambar S, Kulkarni S, Armstrong S, Nikolarakos D (2020). Botulinum toxin in the management of temporomandibular disorders: a systematic review. Br J Oral Maxillofacial Surg.

[19] Safarpour Y, Jabbari B (2018). Botulinum toxin treatment of pain syndromes–an evidence based review. Toxicon.

[20] Li T, Liu Y, Zhang W (2018). Botulinum toxin A plays an important role in the placement of implants deep within the pectoralis major muscle for mammaplasty: a systematic review and meta-analysis. Aesthetic Plast Surg.

[21] Yi K-H, Lee H-J, Seo KK, Kim H-J (2022). Botulinum neurotoxin injection guidelines regarding flap surgeries in breast reconstruction. J Plast Reconstruct Aesthetic Surg.

[22] Winocour S, Murad MH, Bidgoli-Moghaddam M, Jacobson SR, Bite U, Saint-Cyr M, Tran NV, Lemaine V (2014). A systematic review of the use of Botulinum toxin type A with subpectoral breast implants. J Plast Reconstruct Aesthetic Surg.

[23] Moher D, Liberati A, Tetzlaff J, Altman DG (2009) Preferred reporting items for systematic reviews and meta-analyses: the PRISMA statement. BMJ 339 PMC3090117 21603045

[24] Higgins JPT, Green S (eds) (2008) Cochrane Handbook for Systematic Reviews of Interventions. Cochrane Collaboration

[25] WebPlotDigitizer RA. 4.3, (2020)

[26] Higgins JP, Altman DG, Gøtzsche PC, Jüni P, Moher D, Oxman AD, Savović J, Schulz KF, Weeks L, Sterne JA (2011). The cochrane collaboration’s tool for assessing risk of bias in randomised trials. BMJ.

[27] National Heart Lung, and Blood Institute (2014) Quality assessment tool for observational cohort and cross-sectional studies. Bethesda: National Heart, Lung, and Blood Institute

[28] Hozo SP, Djulbegovic B, Hozo I (2005). Estimating the mean and variance from the median, range, and the size of a sample. BMC.

[29] Higgins JP, Thompson SG, Deeks JJ, Altman DG (2003). Measuring inconsistency in meta-analyses. BMJ.

[30] Borenstein M, Hedges L, Higgins J, Rothstein HJRJ (2005) Comprehensive meta-analysis V2 [Computer software and manual] 24:2007

[31] Copenhagen DTNCC, Cochrane Collaboration (2014) Review manager (version 5.4) [computer software]

[32] Abedini R, Mehdizade Rayeni N, Haddady Abianeh S, Rahmati J, Teymourpour A, Nasimi M (2020). Botulinum toxin type A injection for mammoplasty and abdominoplasty scar management: a split-scar double-blinded randomized controlled study. Aesthetic Plast Surg.

[33] de Carlos IE, Hedo AC, González MM, Caravaca GR, Ruiz-Soldevilla J, Pérez BS (2012). Infiltración con toxina botulínica para el control del dolor en cáncer de mama. Rehabilitación.

[34] De Groef A, Devoogdt N, Van Kampen M, Nevelsteen I, Smeets A, Neven P, Geraerts I, Dams L, Van der Gucht E, Debeer P (2018). Effectiveness of botulinum toxin A for persistent upper limb pain after breast cancer treatment: a double-blinded randomized controlled trial. Arch phys Med Rehabil.

[35] Disphanurat W, Viarasilpa W, Thienpaitoon P (2021). Efficacy of botulinum toxin a for scar prevention after breast augmentation: a randomized double-blind intraindividual controlled trial. Dermatol Surg.

[36] Ermilova E, Zinovev E, Yampolskaya E (2020). Treatment of pain syndrome after aesthetic breast surgery with botulinum toxin type A. Innovative Med Kuban.

[37] Figus A, Mazzocchi M, Dessy LA, Curinga G, Scuderi N (2009). Treatment of muscular contraction deformities with botulinum toxin type a after latissimus dorsi flap and sub-pectoral implant breast reconstruction. J Plast Reconstruct Aesthetic Surg.

[38] Gabriel A, Champaneria MC, Maxwell GP (2015). The efficacy of botulinum toxin A in post-mastectomy breast reconstruction: a pilot study. Aesthetic Surg J.

[39] Layeeque R, Hochberg J, Siegel E, Kunkel K, Kepple J, Henry-Tillman RS, Dunlap M, Seibert J, Klimberg VS (2004). Botulinum toxin infiltration for pain control after mastectomy and expander reconstruction. Ann Surg.

[40] Lemaine V, Lohse CM, Mandrekar JN, Ramaker SA, Convery PA, Nguyen MD, Tran NV (2020) Botulinum toxin A in tissue expander breast reconstruction: a double-blinded randomized controlled trial. Plast Reconstruct Surg Global Open 8(8) 10.1097/GOX.0000000000003030 PMC7489665 32983785

[41] Lo KK, Aycock JK (2015). A blinded randomized controlled trial to evaluate the use of botulinum toxin for pain control in breast reconstruction with tissue expanders. Ann Plast Surge.

[42] Ma IT, Yesantharao P, Darrach HM, Seither JG, He H, Nguyen DH (2021). Diagnostic and therapeutic use of botox for breast reconstruction. Arch Clini Med Case Rep.

[43] Puentes-Gutiérrez A-B, García-Bascones M, Sánchez-Casado M, Fernández-García L, Puentes-Gutiérrez R, Marquina-Valero M-A (2021) Treatment of post-breast surgery pain syndrome with botulinum toxin: analysis of the response to the addition of levobupivacaine and to the type of surgery. Arch Breast Cancer 8(2):115–8

[44] Dressler D, Johnson EA (2022) Botulinum toxin therapy: past, present and future developments. J Neural Transm 129(5–6):829–33 10.1007/s00702-022-02494-5 PMC9188496 35396965

[45] Datta Gupta A, Edwards S, Smith J, Snow J, Visvanathan R, Tucker G, Wilson D (2022). A systematic review and meta-analysis of efficacy of botulinum toxin a for neuropathic pain. Toxins.

[46] Wang W, Liu G, Li X (2022) The efficacy and safety of botulinum toxin type A injections in improving facial scars: a systematic review and meta-analysis. Pharmacol 107(5–6):241–9 10.1159/000522396 35354154

[47] Yue S, Ju M, Su Z (2021) A systematic review and meta-analysis: botulinum toxin A effect on postoperative facial scar prevention. Aesthetic Plast Surg 46:395–405 10.1007/s00266-021-02596-7 34609526

[48] Lee B-J, Jeong J-H, Wang S-G, Lee J-C, Goh E-K, Kim H-W (2009). Effect of botulinum toxin type a on a rat surgical wound model. Clin Exp Otorhinolaryngol.

[49] Park GS, An MK, Yoon JH, Park SS, Koh SH, Mauro TM, Cho EB, Park EJ, Kim KH, Kim KJ (2019). Botulinum toxin type a suppresses pro-fibrotic effects via the JNK signaling pathway in hypertrophic scar fibroblasts. Arch Dermatol Res.

[50] Gassner HG, Sherris DA, Otley CC (2000). Treatment of facial wounds with botulinum toxin a improves cosmetic outcome in primates. Plast and Reconstruct Surg.

[51] Yi K-H, Lee H-J, Choi Y-J, Lee J-H, Hu K-S, Kim H-J (2020). Intramuscular neural distribution of rhomboid muscles: evaluation for botulinum toxin injection using modified Sihler’s method. Toxins.

